# Audit of therapeutic drug monitoring of ‘clozapine plasma levels’

**DOI:** 10.1192/bjo.2021.821

**Published:** 2021-06-18

**Authors:** Shakina Bellam, Martina Khundakar, Priya Khanna

**Affiliations:** 1 Monkwearmouth Hospital, Sunderland, Cumbria, Northumberland Tyne Wear NHS Foundation Trust; 2 St Nicholas Hospital, Cumbria Northumberland Tyne Wear NHS Foundation Trust; 3 Palmer's Community Hospital, Cumbria Northumberland Tyne Wear NHS Trust

## Abstract

**Aims:**

To re audit the monitoring of Plasma Clozapine levels in Rehabilitation setting in CNTW Trust as per Trust Guidelines PGN on “Safe prescribing of Clozapine”.

Objectives:

To determine if

1. The reason for a clozapine plasma level request is recorded.

2. Results are recorded correctly.

3. Appropriate action is taken and recorded when results are significant.

**Background:**

Clozapine plasma level monitoring is useful when assessing adherence, adjusting the dose, monitoring the effects of changes in smoking habit, investigating clozapine side effects and when toxicity is suspected.

An initial audit was carried out within the Trust in 2015 and the following recommendations were made:

Check and record clozapine plasma level

At baseline (a level should be taken once the patient has been on the target dose for at least a week)

Annually.

When clinically relevant to optimise therapy.

An entry must be made in the patient's progress notes recording the reason of requesting the test.

On receipt of results, the paper copy must be scanned & an entry made in progress notes.

The clinician should comment on the significance of the results and propose an action plan.

We re-audited compliance with the guidance in Rehabilitation (inpatients and community) by reviewing patient notes for a 2 year period of 2017–2018.

**Method:**

The audit work involved a review of 31 case records of patients prescribed Clozapine whose last plasma level was taken between 2017–2018. Patient's details were identified from a randomly generated list by the Trust pharmacy.

**Result:**

<50% compliance was seen with baseline, annual monitoring, reason for recording and proposed action plan by clinician.

>50% compliance was seen with scanned results and levels checked when clinically relevant.

No significant improvement from the previous audit except improvement in compliance with documentation of levels.

**Conclusion:**

Dissemination of Clozapine Key cards within teams.

